# A case of resistant scleral thinning following uneventful pterygium surgery: A case report and a literature review

**DOI:** 10.1016/j.ijscr.2022.107223

**Published:** 2022-05-18

**Authors:** Dana Sultan, Othman Alnema, Mohamad Nour Sultan, Nadim Zahlouk, Ammar Kayyali

**Affiliations:** a Department of Ophthalmology, Aleppo University Hospital, Aleppo, Syria; b Aleppo University Faculty of Medicine, Aleppo, Syria

**Keywords:** Pterygium, Scleral dellen, Thinned sclera, Conjunctival autograft, Case report, SINS, Surgically Induced Necrotizing Scleritis, SD, Scleral Dellen

## Abstract

**Introduction:**

We describe the management of a scleral thinning after uneventful pterygium surgery, it is an uncommon complication; in addition, we have reviewed similar published cases in the literature.

**Presentation of case:**

A 48-year-old woman presented with thinning sclera in the first week after pterygium excision surgery. Conservative treatment was the first line in the management. There was no improvement for two weeks, so we decided to do a rotational flap. We put a scleral dellen diagnosis by excluding other etiologies. The condition was successfully managed, the thinning sclera healed completely. Scleral and conjunctival re-surfacing was observed.

**Clinical discussion:**

Scleral dellen is an early and rare postoperative complication after pterygium surgery. The diagnosis is confirmed after excluding other causes of scleral thinning. The exact pathophysiology of it is not determined yet, many authors described probable explanation in their published cases. We did a comprehensive review of similar cases with their management.

**Conclusions:**

Scleral dellen is uncommon complication after pterygium surgery, its diagnosis depends on exclusion. The management can be conservative. However, if no progression was detected do not hesitate going for surgical closure.

## Highlights

• We describe the management of scleral thinning after uneventful pterygium surgery.
• Scleral dellen is uncommon complication after pterygium surgery.
• The diagnosis of Scleral dellen is confirmed after excluding other causes of scleral thinning.
• The management can be conservative. However, if no progression was detected, surgical closure is the final management.

## 1 Introduction

Pterygium is a fibrovascular growth of the conjunctiva on the corneal surface. Several surgical techniques are available for the treatment of pterygium; excising accompanied by adjunctive treatment with mitomycin C or conjunctival autografts is currently the most popular treatment option since it provides the most satisfactory results [1], [2].

The condition of scleral defect after pterygium surgery has been reported in many publications as a scleral dellen, scleral melting, scleral thinning and scleral necrotizing defects. In addition, there is not a definitive pathology described for this complication neither guidelines for treatment.

In this case, we present a severe acute scleral defect occurred after an uneventful pterygium surgery. In addition, we reviewed similar published cases and their management. This work has been reported in line with the SCARE criteria [3].

## 2 Case report

A 48-year-old Caucasian woman presented to the outpatient clinic with a history of recurrent irritation, redness and foreign body sensation in her right eye. Ophthalmologic examination revealed a primary pterygium on the nasal side of her right eye, measuring 3 mm at the limbus and 4 mm into the cornea. No other abnormalities were detected in the eye. She denied any systemic, ocular or medical histories. The pterygium was excised by a senior resident under topical and subconjunctival anesthesia (lidocaine 2% and epinephrine 1/200,000). A conjunctival autograft was performed to cover the conjunctival defect using sutures without any adjunctive therapies such as intraoperative mitomycin C or cauterization, nor postoperative beta radiation.

The operation ended smoothly. We gave the patient a combination therapy of levofloxacine 0.5% and dexamethasone 0.1% eye drops four times daily after surgery with antibiotic ointment.

The outcome looked satisfactory the day after surgery without any complaints or complications. Three days later, however, the graft shrank and appeared grey and dry. Areas of bare sclera have appeared around the graft with congested conjunctiva. After asking her again, she denied any history for medical, ocular conditions or taking medications. An extensive therapy of topical steroids, antibiotics, and artificial tears was given.

**Fig. 1:**
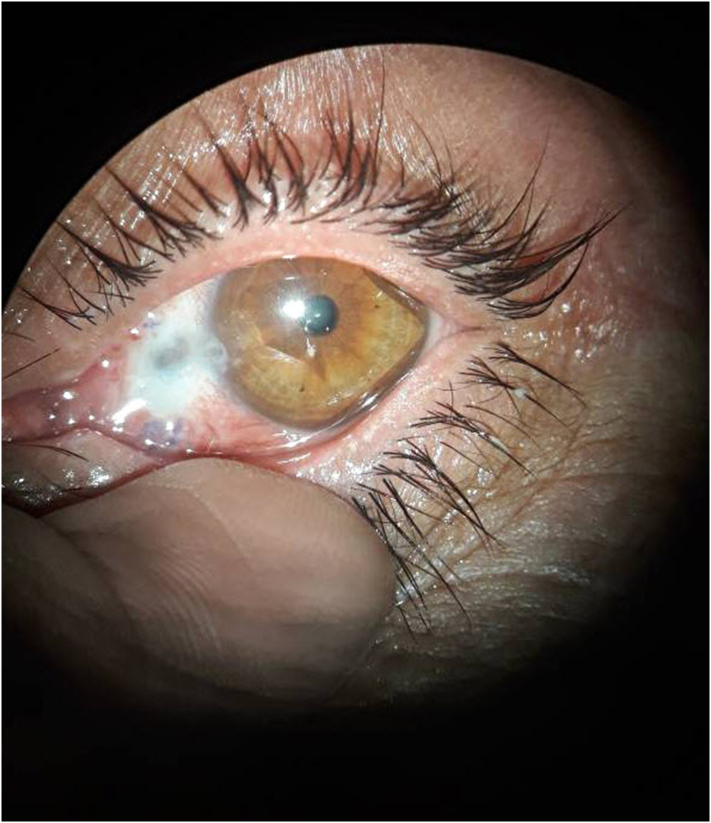
Shows the defect after a week of the surgery.

One week later, the patient came to the department complaining of mild discomfort, foreign body sensation and a black dot at the surgical site. Her corrected visual acuity was 20/25 just as before the operation, the intraocular pressure was 16 mm Hg. On slit lamp examination, the conjunctival sutures were no longer in place and a well-demarcated focal area of scleral thinning was visible measuring approximately 2 ∗ 2.5 mm surrounded by congested edematous conjunctiva. The thinned sclera was dry and the uvea was visible through the base of the lesion (Fig. 1). There was no ciliary injection, no papillary or follicular reaction at the palpebral conjunctiva. No cells or flair in the anterior chamber, B-scan was normal. The rest of the eye exam was normal.

The patient was referred to the Internal Medicine Department in order to rule out autoimmune or any infectious diseases. Medical history, physical examination, and laboratory tests (blood count, biochemistry, rheumatoid factor and antinuclear antibody screening) were all in normal ranges.

We made a diagnosis of scleral dellen and continued the mentioned medications with hourly lubrication. After two weeks of conservative treatment, scleral thinning did not decrease in size despite the intensive ocular lubrication in the treatment.

Finally, we decided to do a surgical management with a rotation flap of adjacent conjunctiva.

**Fig. 2:**
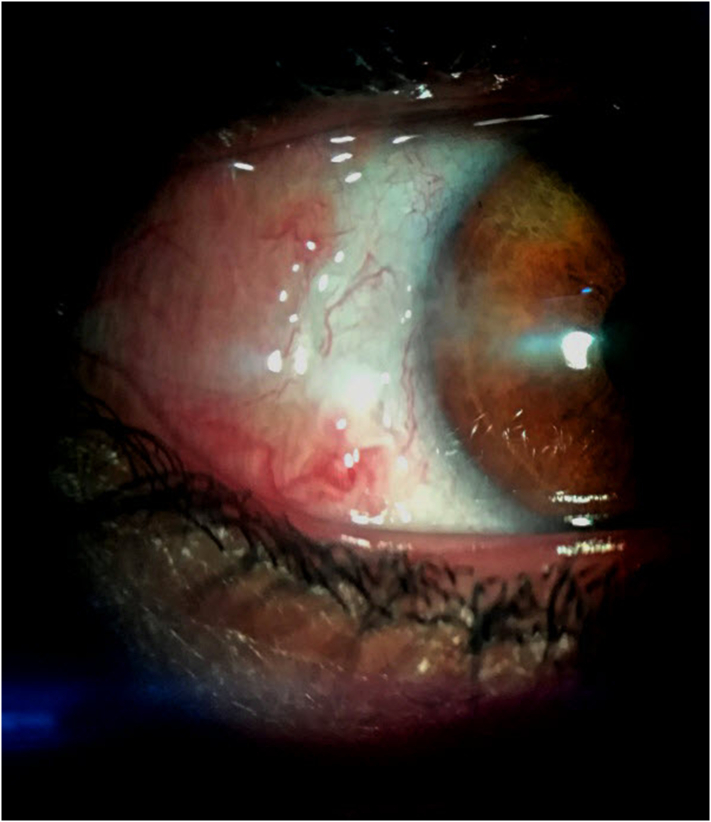
One month after the conjunctival flap procedure.

The medication regime contained levofloxacin 0.5% and dexamethasone 0.1% eye drops four times daily with unpreserved artificial tears 24 times a day after surgery. The outcome was good as the patient symptomatically improved and the thinning area decreased in size. Four weeks after the procedure, the thinned sclera appeared regularly thick and white in color with no more visible uvea (Fig. 2).

## 3 Discussion

We are presenting a challenging case of scleral thinning after uneventful pterygium excision with autograft that covered the conjunctival defect without any adjunctive therapies.

Scleral dellen is an early postoperative complication during first two weeks of bare sclera technique owing to delayed conjunctival wound closure [4], [5]. The formation of a dry area on the cornea, limbus even sclera is not uncommon which could lead to the formation of dellen. The exact pathophysiology of this complication on sclera is not determined [5], [6]. Chen and Noonan [6] in addition to T. Starck et al. [7] suggest that conjunctival edema with raised granulation tissue edges may cause a discontinuity of the tear film leading to exacerbated local desiccation and dellen formation. Tsai et al. [5] added that the delayed conjunctival wound closure should be taken as another factor. In Accorinti et al. case [8], authors assumed that tear film stability disruption and dehydration may also contribute to dellen formation. On the other hand, S. Mitra et al. [9] suggest a contribution of steroids in the pathology of dellen development.

Our first differential diagnosis to rule out was surgically induced necrotizing scleritis (SINS); SINS is a painful condition, patients typically offer history of serious, profound boring pain. In addition, the majority of cases of SINS present within few months of surgery. The managment of the thinned sclera is difficult [10].

Our patient however, was healthy at presentation. She had not any sever pain or symptoms. There are not any conditions predispose to ulceration, poor wound healing or severe tear film. Schirmer-Test was within normal limits. All laboratory studies were normal, no systemic diseases such as autoimmune processes or vasculitis were detected at presentation. Apart from the drying effect produced by the edematous conjunctiva and the loss of the graft, no other etiological factor was found.

We did a comprehensive review of similar cases with their management; we found 12 published articles. All of them, are case reports or case series. The results are concluded in Table 1. Half of the published cases were healed with the conservative treatment along with patching. However, the other half needed a surgical intervention.

**Table 1:** Scleral dellen cases after uneventful pterygium surgery published in the literature.

The case	Surgery	Onset of symptoms	Intraoperative adjunctive therapy	Management	Results after surgery
S. Chen, C. Noonan [6]	Bare sclera excision	14 days	Minimum cauterization	Conjunctival flap	Not mentioned
S. Mitra et al. [9]	Bare sclera technique	7 days	Not mentioned	Discontinued steroid, patching for 24 h then artificial tears 2 hourly	Complete resolution in the next 6 day
Tsai et al. [5]	Bare sclera technique	8 days	Mitomycin C	Discontinued steroid, ointment ciprofloxacin was applied with intensive artificial tears	The area appeared normal 3 days later
Hicks et al. [11]	Bare sclera	2 days	Beta-radiation	Patching initially for 45 min then until recover	Not mentioned
Safianik B. et al. [12]	Bare sclera	21 days	Case 2: Mitomycin C	Unresponsive to conservative treatment, autologous conjunctival graft surgery was done	2 weeks later the graft showed good adaptation and re-epithelialization
		21 days	Case 3: Mitomycin C	Unresponsive to conservative topical treatment, then conjunctival flap	Re-epithelialization
Garcia-Medina et al. [13]	Simple conjunctival closure	7 days	Minimum cauterization	Patching, antibiotic, and artificial tears	Defect healed within a few weeks
A.Kurt et al. [14]	Conjunctival auto- graft	11 days	Minimum cauterization	Topical autologous serum at 30% concentration hourly.	Complete epithelialization on the postoperative 22nd day
M. Accorinti et al. [8]	Bare scleral technique	14 days	Not mentioned	Discontinuing topical steroids. Add intensive lubricants, tetracycline topical antibiotic, patching and 3 tablets daily of a mix of L-amino acids	One week later, the corneal dellen had completely healed and, 4 weeks later, the thinned sclera appeared regularly thick
A. Agarwal, D. A. Kumar [15].	Conjunctival autograft	7 days	None	Scleral patch grafting	Healed
MS Sridhar, AK Bansal and GN Rao [16]	Not mentioned	6 days	Not mentioned	Two cases, in both antibiotics along with artificial tears were prescribed. The patient underwent multilayered amniotic membrane transplantation over the area of scleral thinning.	Epithelialization in 6 weeks.
Chavhan P, Stephen MBabu K et al. [17]	Conjunctival autograft	Two weeks	None	Scleral patch graft	After 3 months complete epithelialization
Bade Ogundipe et al. [18]	Bare sclera	7 days	Beta irradiation	Corneo-scleral patch	Improved after one month.

A conservative treatment consisting of the use of extensive artificial tears, antibiotic ointment and a patch is a useful management for the major of scleral dellen cases. However, if the case does not respond to the treatment, a surgical approach should be considered. Conjunctival flap and multilayered amniotic membrane transplantation are useful operations with minimum complications. In addition, when the case is associated with an Impending perforation, a lamellar scleral patch graft may be undertaken.

More observational and case-control studies are needed in order to investigate the pathophysiology of this condition and to describe guidelines for the management.

## 4 Conclusion

Conservative treatment is an appropriate initial treatment for scleral dellen. In resistant cases, conjunctival flap may be a good management. More studies on the histopathology and pathophysiology should be done to identify the actual ground for this complication.

## Consent

Written informed consent was obtained from the patient for publication of this case report and accompanying images. A copy of the written consent is available for review by the Editor-in-Chief of this journal on request.

## Provenance and peer review

Not commissioned, externally peer-reviewed.

## Ethical approval

Case reports are exempt from ethnical approval in our institution.

## Funding

This article does not have funding sources.

## Guarantor

Dr. Dana Sultan.

## Research registration number

N/A.

## CRediT authorship contribution statement

**DS**: Is the first author, Conceptualization, Validation, Investigation, Writing - Original draft, Writing Review & editing, Visualization. **ON**: The surgeon, Conceptualization, Writing - Original draft, Writing - Review & editing, Investigation. **MNS**: Data curation, Writing - Review & editing, Visualization. **NZ and AK**: Supervision, Writing - Review & editing, Project administration. All authors attest that they meet the current ICMJE criteria for Authorship.

## Declaration of competing interest

All authors have no conflict of interest to declare.
